# New view in cell death mode: effect of crystal size in renal epithelial cells

**DOI:** 10.1038/cddis.2015.359

**Published:** 2015-12-10

**Authors:** X-Y Sun, J-M Ouyang

**Affiliations:** 1 Department of Chemistry, Jinan University, Guangzhou, China

In 2009, the Nomenclature Committee on Cell Death proposed a set of recommendations for the definition of distinct cell death morphologies and for the appropriate use of cell death-related terminology, including ‘apoptosis', ‘necrosis' and ‘mitotic catastrophe'.^1^ Of which, apoptosis and necrosis is always the most concern topic on cell death mode to researchers. Apoptosis has come to be used synonymously with the phrase ‘programmed cell death' as it is a cell intrinsic mechanism for suicide that is regulated by a variety of cellular signaling pathways. During apoptotic death, cells are neatly carved up by caspases and packaged into apoptotic bodies as a mechanism to avoid immune activation. In contrast to apoptosis, necrosis has been traditionally thought to be a passive form of cell death with more similarities to a train wreck than a suicide.^2^

Kidney stones are a common and frequently occurring disease, >70% of kidney stone patients suffer from urolithiasis caused by calcium oxalate (CaOx) stones, but the mechanism by which kidney stones are formed has not yet been completely clarified. Especially, the mode of cell death produced by CaOx is confusing to us, by far. Saha *et al.*^3^ have demonstrated that exposure of cells to CaOx crystals can lead to significant apoptotic changes, including condensation and margination of nuclear chromatin, DNA fragmentation, and migration of phosphatidylserine (PS) of the plasma membrane from inside the cell membrane to the cell surface. However, Schepers *et al.*^4^ have proven that exposure of cells to CaOx crystals results in necrotic cell death with significant necrotic changes, such as loss of plasma membrane integrity, release of lactate dehydrogenase, cellular and nuclear swelling, and inflammatory response. In general, fast-acting metabolic poisons and strong physical stress, such as freezing, boiling, or shearing, rupture cell membranes and cause rapid cell necrosis. By contrast, a slow acting form of cell death called apoptosis does not involve membrane damage and inflammation.^5^ Cell death is a complicated and confusing pathological process. Cell apoptosis and necrosis caused by CaOx crystal exposure may be related to cell types, crystal concentration, exposure time, and even the unknown physicochemical properties of crystals.

In our new paper in *Cell Death Discovery*,^6^ we comparatively investigated the differences of cell death mode induced by nano-sized (50 nm) and micron-sized (10 *μ*m) calcium oxalate monohydrate (COM) and dihydrate (COD) to explore the cell death mechanism. Exposure to nano-/micron-sized COM and COD crystals triggered both apoptotic and necrotic cell death in renal epithelial cell lines. However, nano-sized crystals primarily caused apoptotic cell death, leading to cell shrinkage, PS ectropion, and nuclear shrinkage, whereas micron-sized crystals primarily caused necrotic cell death, leading to cell swelling and cell membrane and lysosome rupture.

The cell death mechanism induced by nano-/micron-sized COM and COD is summarized in the schematic in Figure 1. Nano-sized COM and COD crystals are more likely to be internalized by cells than micron-sized crystals. These internalized nano-sized crystals were transferred into lysosomes via vesicular transport, and could be degraded in lysosomes to release calcium and oxalate ions. Internalized nano-sized crystals may increase the lysosomal membrane permeability in varying degrees. A prevalent assumption is that the reparable damage of lysosomes can initiate apoptosis, and a sudden massive destruction of lysosomes leads to necrosis.^7^ The nano-sized crystals were evenly distributed on cell surface; they induced mild injury instead of partial acute injury. After the treatment by nano-sized crystals, the cells and nuclear shrank, presenting typical apoptosis characteristics. The mitochondria were seriously injured and the mitochondrial membrane potential was significantly decreased. Apoptotic cells maintained their plasma membrane integrity, but PS was translocated on the cell membrane. The internalized crystals could not only be captured by lysosomes, but also entered into the nucleus through the nuclear pores, leading to the cleavage of DNA into internucleosomal fragments of 180 bp and multiples, which is an important characteristic of apoptotic cell death.

For micron-sized COM and COD crystals, their particle number is much less than that of nano-sized crystals under the same concentration. Micron-sized crystals on cells presented a nonhomogeneous distribution, which caused uneven injury of the cell membrane and local strong physical stress, resulting in necrotic cell death. Necrotic cells released inflammatory factors and led to cell membrane rupture. Cell membrane rupture can cause an imbalance in cell osmotic pressure and lead to the sudden massive destruction of lysosomes accompanied with hydrolytic enzyme release, which is an important factor in necrotic cell death.^8^ Meanwhile, sudden massive release of hydrolytic enzyme could lead to the random degradation of chromatin DNA, resulting in necrotic cell death.

Cell apoptosis induced by nano-sized COM and COD was accompanied with PS ectropion, whereas necrosis induced by micron-sized crystals was not. This exposed negatively charged PS acted as a binding site of urine microcrystalline on the cell surface and increased the adhesion and aggregation of microcrystallines, thereby increasing the risk of stone formation. Therefore, compared with necrosis, apoptosis may be more likely to induce stone formation. In general, stone formers tend to excrete urine that is more supersaturated than that of non-stone formers. The median size of initial formed crystals is inversely related to relative supersaturation,^9^ therefore, the initially formed urinary crystallites in stone formers would be smaller than that in healthy controls. Thus, the higher supersaturated urine in stone formers should be more likely to induce apoptotic cell death, which will increase the adhesion and aggregation of microcrystallines and more easily lead to stone formation.

Besides, crystal shape, crystal structure, and even other physical and chemical properties may also affect the mode of cell death, but the relevant studies were very limited. Braydich-Stolle *et al.*^10^ reported that crystal structure of TiO_2_ could mediate the cell death mode in mouse keratinocyte cells. The anatase TiO_2_ nanoparticles induced cell necrosis, while the rutile TiO_2_ nanoparticles initiated apoptosis through the formation of ROS. However, in our present study,^6^ the mode of cell death produced by the same-sized COM and COD crystals has no obvious difference. Therefore, cell death is a complicated pathological process, the detailed effect of physicochemical properties of crystals in cell death mode still calls for further research.

## Figures and Tables

**Figure 1:**
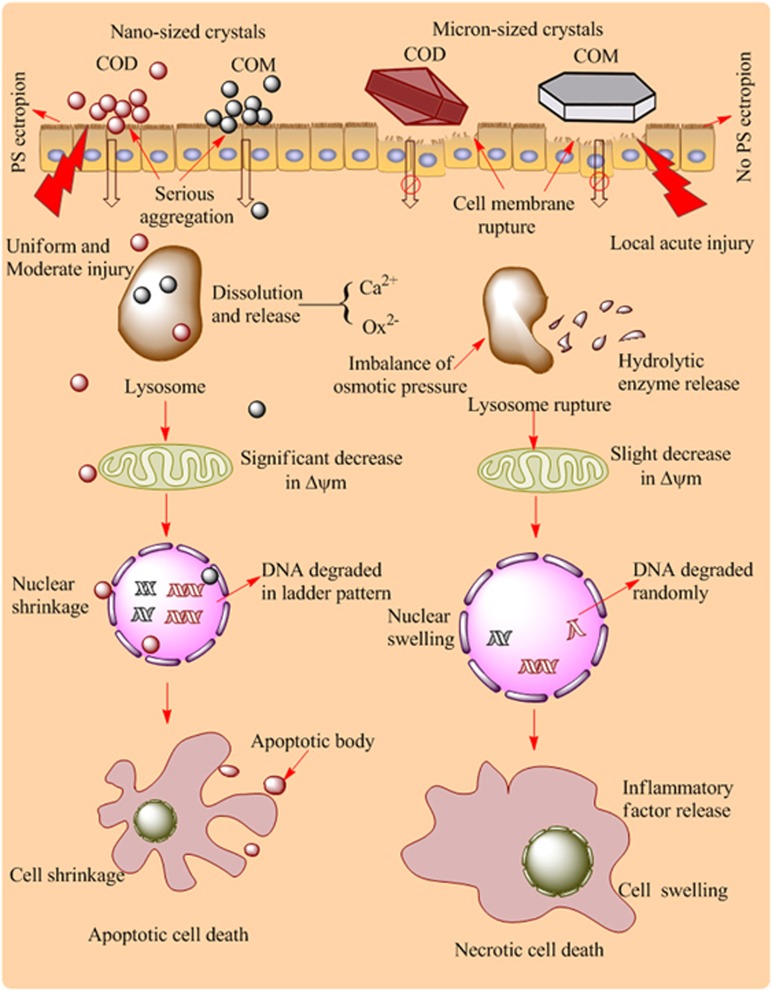
The mediation of calcium oxalate size on cell death mode in Vero cells. Nano-sized COM and COD crystals primarily caused apoptotic cell death owing to their small size effect and uniform and moderate injuries. Micron-sized crystals primary caused necrotic cell death due to their large size and local acute injuries
